# Advanced non-cardia gastric cancer and *Helicobacter pylori* infection in Vietnam

**DOI:** 10.1186/s13099-017-0195-8

**Published:** 2017-08-17

**Authors:** Tran Thanh Binh, Vo Phuoc Tuan, Ho Dang Quy Dung, Pham Huu Tung, Tran Dinh Tri, Ngo Phuong Minh Thuan, Vu Van Khien, Phan Quoc Hoan, Rumiko Suzuki, Tomohisa Uchida, Tran Thi Huyen Trang, Yoshio Yamaoka

**Affiliations:** 1 Department of Environmental and Preventive Medicine, Oita University Faculty of Medicine, 1-1 Idaigaoka, Hasama-machi, Yufu, Oita 879-5593 Japan; 2 Department of Endoscopy, Cho Ray Hospital, Ho Chi Minh, Vietnam; 3 Department of Hepatogastroenterology, 108 Military Central Hospital, Hanoi, Vietnam; 4 Department of Molecular Biology, 108 Military Central Hospital, Hanoi, Vietnam; 5 Department of Molecular Pathology, Oita University Faculty of Medicine, Yufu, Japan; 6 Department of Medicine-Gastroenterology, Baylor College of Medicine, Houston, TX USA

**Keywords:** Gastric cancer, *Helicobacter pylori*, Virulence factor, *vacA*, Diffuse type, Intestinal type

## Abstract

**Background:**

The incidence of gastric cancer in the Northern city, Hanoi is higher than in the Southern city, Ho Chi Minh, Vietnam. We previously reported that *Helicobacter pylori vacA* m1 genotype might be responsible for the difference between the two cities, however, the study only included non-cancer patients. The aim of this study is to investigate the non-cardia gastric cancer characteristics and the role of *H. pylori* virulence on different non-cardia gastric cancer incidence between two cities in Vietnam.

**Methods and Results:**

We recruited 282 non-cardia gastric cancer patients that had undergone gastroscopy in two cities, Ho Chi Minh and Hanoi, Vietnam. Characteristics of non-cardia gastric cancer were late age of onset (mean age, 62.5 years), predominance in males (ratio of males/females; 3.9:1), diffuse type (55.3%), and high prevalence of *H. pylori* infection (79.4%). *H. pylori* infection and the *vacA* m1 genotype conferred an increased risk for GC (OR, 2.02; 95% CI 1.4–3.0; *P* = 0.0003 and OR, 2.7; 95% CI 1.5–4.7; *P* = 0.001, respectively). Interestingly, the presence of *vacA* m1 genotype in the gastric cancer group was significantly higher than that in the non-cancer group (68.8% vs 44.9%, *P* = 0.001) and the significant tendency still observed in Ho Chi Minh (67.6% vs 31.9%, *P* < 0.0001).

**Conclusion:**

We first describe the characteristics of non-cardia gastric cancer in Vietnam. *Helicobacter pylori* infection was associated with the development of non-cardia GC. *vacA* m1 genotype might contribute to incidence differences between the two cities.

**Electronic supplementary material:**

The online version of this article (doi:10.1186/s13099-017-0195-8) contains supplementary material, which is available to authorized users.

## Background

Gastric cancer (GC) is the fifth most common cancer worldwide and the third most common cancer in Asia (GLOBOCAN 2012; http://globocan.iarc.fr). Although GC incidence and mortality rates have been slowly declining over the last few decades in most countries, GC still remains a significant public health problem [1]. In Asia, where GC is the third most common cause of cancer death, the incidence and mortality of GC vary widely among countries, even among different ethnic groups or regions in the same country [2–4]. Etiologically, GC is closely associated with many factors such as age, dietary and lifestyle factors, genetics, and especially *Helicobacter pylori* (*H. pylori*) infection and its virulence factors [4–6].

*Helicobacter pylori* is a spiral, gram-negative bacterium that has colonized the stomachs of approximately half of the world’s population. *H. pylori* infection is the strongest risk factor for GC, and most *H. pylori*-infected people develop chronic gastritis [7–10]. However, only a small proportion of infected subjects has a potentially higher risk of developing non-cardia or distal GC (antrum or corpus) [9]. One reason underlying this observation is the difference in bacterial pathogenicity. *H. pylori* strains are genetically highly diverse, and several genes/genotypes are strongly associated with virulence and are consequently linked to carcinogenicity. Among virulence factors, the diverse clinical outcomes of *H. pylori* infection are strongly associated with the diversity of *cagA* and *vacA* [8, 11].

CagA is the most widely studied *H. pylori* virulence factor [12]. More than 90% of *H. pylori* isolated from East Asian countries carry *cagA*, whereas only 50–70% of *H. pylori* isolated from Western countries do [13, 14]. The CagA protein has been classified into Western-type and East Asian-type based on the sequences of repeat regions of CagA containing Glu-Pro-Ile-Tyr-Ala (EPIYA) motifs [12, 13]. Individuals infected with East Asian-type CagA *H. pylori* have been reported to have an increased risk of peptic ulcer disease (PUD) and/or GC, compared to those infected with Western-type CagA strains [15–17].

VacA is another extensively studied *H. pylori* virulence factor [12]. The vacuolating activity varies among different *H. pylori* strains [18] because of the different structure of *vacA*, which consists of the signal (s) (s1 and s2) and the middle (m) (m1 and m2) regions [19]. It has been showed that *vacA* s1m1 exhibits the most cytotoxicity, followed by *vacA* s1m2, whereas *vacA* s2m2 does not display any cytotoxicity [19]. Furthermore, individuals infected with *vacA* s1 or m1 *H. pylori* have an increased risk of PUD and/or GC compared to those infected with s2 or m2 strains [19, 20].

Vietnam has emerged as a country with the highest age-standardized incidence rate (ASR) of GC (16.3 cases/100,000 for both sexes) in Southeast Asia (GLOBOCAN 2012; http://globocan.iarc.fr). In keeping with the consensus, our previous studies have reported the high prevalence of *H. pylori* infection in Vietnam and its strong association with PUD, active gastritis, atrophy, and intestinal metaplasia [21]. However, these studies were from a short-term, cross-sectional design study, which is why the samples included only gastritis and PUD. Therefore, in this study, we aimed to exclusively study non-cardia GC subjects in order to describe the association between *H. pylori* infection and non-cardia GC, as well as characteristics about demographics and histopathology of non-cardia GC in Vietnam. In addition, statistical data indicate that the ASR of GC in Hanoi, the capital city located in the north, is approximately 1.5 times higher than that in Ho Chi Minh, the second largest city located in the south. This phenomenon was called the “Vietnam enigma” [22]. In an effort to clarify this phenomenon, our previous study included gastritis and PUD patients, but not GC patients, and showed that the *vacA* m1 type was associated with an increased risk for PUD and was significantly more common in Hanoi (54%) than in Ho Chi Minh (31%) [21]. We, therefore, suggested that the *vacA* m1 type might contribute to the difference in the incidence of GC between these two cities.

## Methods

### Patients and *H. pylori*

Suspected GC patients undergoing gastroscopy at the two endoscopy centers—Cho Ray Hospital, Ho Chi Minh and 108 Military Hospital, Hanoi from November 2012 to May 2014—were recruited. Exclusion criteria included a history of gastrectomy; previous *H. pylori* eradication therapy; treatment with bismuth-containing compounds, H_2_-receptor blockers, or proton pump inhibitors within 2 weeks of the start of the study; and patients with suspected recurrent Billroth anastomosis cancer, entire stomach tumor, or cardiac tumor. Local ethics approval was obtained from the Ethics Committee of Cho Ray Hospital and 108 Military Hospital, and written informed consent was obtained from all patients. The study was also approved by the Ethics Committee of Oita University Faculty of Medicine, Japan.

Before endoscopy, patients were interviewed by trained medical staff to obtain their medical history and lifestyle factors. Depending on the location of the tumor, at least eight biopsy specimens (three from the normal-looking mucosa at least 2–3 cm apart from the tumor margin in the antrum, one from the upper posterior or anterior wall of the corpus, and four from the tumor margin) were taken. Three specimens from the antrum were used for *H. pylori* culture, a rapid urease test, and histological examination. When the antrum was occupied by the tumor, the specimens were obtained from the lower corpus. The corpus specimen was used for histological examination. Suspected GC cases were confirmed by histological examination. GC was classified into intestinal or diffuse type according to the Lauren classification [23]. Blood samples from all participants were collected on the same day. Serum was separated and frozen at −80 °C until analysis.

### Determination of *H. pylori* status

To maximize the diagnostic accuracy, four different tests were used for the diagnosis of *H. pylori* infection: culture, histology including immunohistochemistry (IHC), a rapid urease test (CLO test, Campylobacter-like organism test, Kimberly–Clark Ballard Medical Products, Roswell, GA, USA), and serum *H. pylori* antibody presence.

*Helicobacter pylori* culture was performed as previously described [21]. For histology, hematoxylin and eosin (HE), Giemsa staining, and IHC with an anti-*H. pylori* polyclonal antibody (Dako, Denmark) were performed, and the updated Sydney system was used, as described previously [24, 25]. For the serologic test, we used an ELISA kit (Eiken Co., Ltd, Tokyo, Japan) to detect anti-*H. pylori* antibody according to the manufacturer’s instructions.

*Helicobacter pylori*-positive status was determined by at least one positive result among four tests. *H. pylori*-negative status was diagnosed if all four tests gave negative results.

### Determination of *cagA* and *vacA* virulence factors

*cagA* status was determined by PCR for the conserved region and by direct sequencing, as described previously [26]. Briefly, *cagA* was amplified by using the primers cagOMF (5′-AGC AAA AAG CGA CCT TGA AA-3′) and cagOMR (5′-AGT GGC TCA AGC TGC TGA AT-3′). The PCR conditions were initial denaturation for 5 min at 94 °C, 35 cycles of amplification step (94 °C for 30 s, 56 °C for 30 s, 72 °C for 30 s), and a final step of 72 °C for 7 min. The amplified fragment was detected by a 1.5% agarose gel electrophoresis using an UV transilluminator. The PCR products were purified using a QIA quick purification kit (Qiagen) and sequenced by using an AB 3130 genetic analyzer (Applied Biosystems, Foster City, CA). The CagA types (East Asian-type CagA and Western-type CagA) were defined according to the flanking sequences of EPIYA motif patterns. Taken together, the strains possessed EPIYA-A, EPIYA-B, and EPIYA-C segments was considered as Western-type CagA and the strains possessed EPIYA-A, EPIYA-B and EPIYA-D segments was considered as East-Asian-type CagA. *vacA* genotyping (s1, s2, m1, and m2) was performed as described previously [19, 27]. Briefly, *vacA* s region was amplified by using the primers VA1-F (5′-ATG GAA ATA CAA CAA ACA CAC-3′) and VA1-R (5′-CTG CTT GAA TGC GCC AAA C-3′); and *vacA* m region was amplified by using the primers VAG-F (5′-CAA TCT GTC CAA TCA AGC GAG-3′) and VAG-R (5′-GCG TCA AAA TAA TTC CAA GG-3′). The amplified fragment was detected by using a 2% agarose gel electrophoresis using an UV transilluminator. Primers for s region yielded a fragment of 259 bp for s1 variants and 286 bp for s2 variants. Primers for m region yielded a fragment of 570 bp for m1 variants and 645 bp for m2 variants. As a control, the data for the *cagA* and *vacA* genotypes of gastritis and PUD patients in our previous study were compared with data in the present study [28].

### Statistical analysis

Chi squared tests, Fisher’s exact tests, independent sample *t* tests, and one-way ANOVA tests were used. Differences at *P* < 0.05 were regarded as statistically significant. Data analysis was performed using SPSS statistical software v.20.0 (SPSS Inc., Chicago, USA).

## Results

### Population study

Among 290 suspected non-cardia GC patients, 282 patients (225 males and 57 females; male/female, 3.9; mean age  ±  standard deviation, 62.5 ± 12.6 years old; age range, 29–87 years) were histologically confirmed as advanced non-cardia GC and were used for analysis. There were no differences in gender distribution by histological subtype (Table 1).

**Table 1:** The characteristics of gastric cancer population based on histologic features

			Lauren type	
	Total	Intestinal type	Diffuse type	Indeterminate
			n (%)	
Total	282	118 (41.8)	156 (55.3)	8 (2.8)
Gender				
Male	225 (79.8)	94 (41.8)	126 (56.0)	5 (2.2)
Female	57 (20.2)	24 (42.1)	30 (52.6)	3 (5.3)
Age groups		64.2 ± 12.3^a^	60.8 ± 12.9^ad^	68.0 ± 8.3
≤39	14 (4.9)	4 (28.6)	10 (71.4)	0 (0.0)
40–49	28 (9.9)	7 (25.0)	21 (75.0)	0 (0.0)
50–59	78 (27.7)	38 (48.7)	39 (50.0)	1 (3.8)
60–69	75 (26.6)	26 (34.7)	45 (60.0)	4 (5.3)
≥70	87 (30.9)	43 (49.4)	41 (47.1)	3 (3.4)
Population group				
Ho Chi Minh	185 (65.6)	68 (36.8)^b^	109 (58.9)	8 (4.3)
Hanoi	97(34.4)	50 (51.5)^abc^	47 (48.5)^a^	0 (0)^c^

^a^Indicates a statistically significant difference between intestinal type vs diffuse type at *P* < 0.05

^b^Indicates a statistically significant difference between Ho Chi Minh vs Hanoi at *P* < 0.05

^c^Indicates a statistically significant difference between intestinal type vs indeterminate type at *P* < 0.05

^d^Indicates a trend of age groups in diffuse type (*P* = 0.022)

There was an increasing trend of intestinal type in the older age group; however, this trend did not reach statistical significance (*P* for trend = 0.081). In contrast, a converse tendency was observed for diffuse type (*P* for trend = 0.022). According to the Lauren classification, the mean age of individuals with intestinal type was significantly higher than that of patients with diffuse type (*P* = 0.027). The population study in Ho Chi Minh showed slightly significant lower age than that in Hanoi (mean age, 61.3 vs 64.5 years old, *P* = 0.045, respectively); however, among the diffuse type groups, there was no statistically different age distribution between Hanoi and Ho Chi Minh (mean age, 59.7 vs 63.2 years old, *P* = 0.13, respectively).

Based on Lauren’s criteria, the prevalence of patients diagnosed with intestinal type was significantly different between Hanoi and Ho Chi Minh (*P* = 0.017), but this was not seen with diffuse type or indeterminate type. Diffuse type cases were found to be predominant in Ho Chi Minh, whereas intestinal type cases were significantly more prevalent than diffuse and indeterminate types among patients in Hanoi (*P* = 0.036 and *P* = 0.021, respectively).

### Prevalence of *H. pylori* infection

The positive *H. pylori* infection rate was 39.7, 42.9, 50.7, and 77.5% in the culture test, histological examination, CLO test, and serologic test, respectively. Given our criteria, in which at least one test yielded positive results, 224 (79.4%) subjects were judged to be infected with *H. pylori*. There was no significant difference in the prevalence of *H. pylori* infection between Hanoi and Ho Chi Minh or between male and female subjects (Table 2). We also failed to find a relationship between *H. pylori* infection and smoking or drinking habits. The infection rate in patients with diffuse type was significantly higher than those with intestinal type (84.6% vs 72.0%, *P* = 0.03) (Table 2). The significantly higher prevalence of infection seen in diffuse type than in intestinal type also was found in Ho Chi Minh (87.2% vs 69.1%, *P* = 0.01). The slightly higher infection rate in diffuse type was also observed in Hanoi (78.7% vs 76%, *P* = 0.9).

**Table 2:** Characteristics of the study population with *Helicobacter pylori* infection

	*H. pylori* positive			*H. pylori* negative	Total
	Total	Past infection	Current infection		
Participants	224 (79.4%)	57 (25.4%)	167 (74.6%)	58 (20.6%)	282
Mean age	62.07 ± 12.32	60.42 ± 10.86	62.63 ± 12.77	63.69 ± 13.89	
City					
Ho Chi Minh	149 (80.5%)	39 (26.2%)	110 (73.8%)	36 (19.5%)	185
Hanoi	75 (77.3%)	18 (24%)	57 (76%)	22 (22.7%)	97
Sex					
Male	177 (78.7%)	46 (25.9%)	131 (74.1%)	48 (21.3%)	225
Female	47 (82.5%)	11 (23.4%)	36 (76.6%)	10 (17.5%)	57
Smoking					
Yes	133 (80.1%)	37 (27.5%)	96 (72.5%)	33 (19.9%)	166
No	90 (82.6%)	20 (22.2%)	70 (77.8%)	19 (17.4%)	109
Drinking					
Yes	124 (81.16%)	35 (28.2%)	89 (71.8%)	18 (18.4%)	152
No	99 (80.5%)	22 (22.2%)	77 (77.8%)	24 (19.5%)	123
Lauren classification					
Intestinal type	85 (72.0%)^a^	20 (23.5%)	65 (76.5%)	33 (28.0%)	118
Diffuse type	132 (84.6%)^a^	35 (26.5%)	97 (73.5%)	24 (15.4%)	156
Indeterminate	7 (87.5%)	2 (28.6%)	5 (71.4%)	1 (12.5%)	8

^a^Indicates a statistically significant difference between intestinal type vs diffuse type at *P* < 0.05

Limitation of serologic test is to detect both current and past infection. Taken together, in order to avoid the false positive results, *H. pylori* positive groups were divided into two groups; current infection and past infection. However, there was no association of age, sex, location, smoking, drinking as well as Lauren classification types between current and past infection (Table 2).

### Distribution of *cagA* and *vacA* genotypes

A total of 112 strains could be isolated, and all strains possessed *cagA* (Table 3). With four distinct CagA EPIYA segments, East Asian-type CagA includes A, -B, and -D segments, while Western-type CagA includes A, -B, and -C segments [27]. Among isolated strains, 110 of 112 (98.2%) strains were East Asian-type, and most of these strains carried ABD type (93.8%), 4 strains (4.2%) had ABB’D, and 1 (1.0%) had BD (B’ is a subgroup of B). There were only 2 of 112 (1.8%) strains with Western-type CagA, and both carried ABC type. The carcinogenic potential of CagA is reported to be linked to its polymorphic EPIYA motif variants [29], and only polymorphisms of the EPIYA motif in B segments were found in this study: EPIYA (93.8%), EPIY**T** (2.7%) and E**S**IYA (3.6%). However, no polymorphism of the EPIYA motif in A and C segments was observed.

**Table 3:** Comparison of the characteristics of patients with *Helicobacter pylori* culture positive between gastric cancer and non-cancer group in Vietnam

	Cancer			Non cancer		
	All cases	Hanoi	Ho Chi Minh	All cases	Hanoi	Ho Chi Minh
Mean age	61.66 ± 13.3^a^	65.02 ± 13.32^b^	59.72 ± 13.98^c^	44.62 ± 13.04^a^	44.28 ± 12.97^b^	45 ± 13.24^c^
Gender						
Male	85 (75.9%)^a^	33 (80.5%)	52 (73.2%)^c^	47 (45.6%)^a^	34 (63%)^d^	13 (26.5%)^cd^
Female	27 (24.1%)	8 (19.5%)	19 (26.8%)	56 (54.4%	20 (37%)	36 (73.5%)
CagA						
Western-type	2 (1.8%)	1 (2.4%)	1 (1.4%)	4 (4.1%)	1 (1.9%)	3 (6.5%)
East Asian-type	110 (98.2%)	40 (97.6%)	70 (98.6%)	94 (95.9%)	51 (98.1%)	43 (93.5%)
Pre-EPIYA						
18 bp deletion-type	94 (85.5%)	33 (84.6%)	61 (85.9%)	80 (77%)	41 (76%)	39 (80%)
39 bp deletion-type	13 (11.8%)	4 (10.2%)	9 (12.7%)	13 (13%)	9 (17%)	4 (8%)
No deletion-type	3 (2.7%)	2 (5.1%)	1 (1.4%)	5 (5%)	3 (6%)	2 (4%)
*vacA* genotype						
s region						
s1	111 (99.1%)	40 (97.6%)	71 (100%)	102 (99%)	53 (98.1%)	49 (100%)
s1s2	1 (0.9%)	1 (2.4%)	0	1 (1%)	1 (1.9%)	0
m region						
m1	77 (68.8%)^a^	29 (70.7%)	48 (67.6%)^c^	44 (44.9%)^a^	29 (56.9%)^d^	15 (31.9%)^cd^
m2	35 (31.2%)	12 (29.3%)	23 (32.4%)	54 (55.1%)	22 (43.1%)	32 (68.1%)

^a^Indicates a statistically significant difference between all GC cases vs all non-GC cases at *P* < 0.05

^b^Indicates a statistically significant difference between GC cases vs non-GC cases in Hanoi at *P* < 0.05

^c^Indicates a statistically significant difference between GC cases vs non-GC cases in Ho Chi Minh at *P* < 0.05

^d^Indicates a statistically significant difference between non-GC cases in Hanoi vs non-GC cases in Ho Chi Minh at *P* < 0.05

Regarding pre-EPIYA repeated region genotypes, 83.9% (94/112) of the strains possessed an 18-bp deletion type, which was reported as the Vietnamese-specific type in our previous study [28], and 11.6% (13/112) strains possessed the 39-bp deletion type, which is called the typical East Asian type. There were no deletion types typically observed in Western-type CagA strains. There was no difference in the pre-EPIYA deletion genotypes between the two cities (Table 3). More detailed analyses of the *cagA* strains are described in Additional file 1.

All strains possessed the *vacA* s1 genotype. The *vacA* m1 genotype was found predominantly in both cities (Table 3). When analyzing the combination of the *vacA* s and m regions, 77 (68.8%) strains were found to be s1m1, 35 (31.2%) were s1m2, and no strains with s2m1 or s2m2 were found. There were no differences between histological GC types and virulence factor genotypes (Table 4).

**Table 4:** The distribution of histological features based on virulence factor genotypes

			Lauren type		
		Total	Intestinal type	Diffuse type	Indeterminate
			n (%)		
Total		112			
CagA					
Western-type		2 (1.8%)	0 (0%)	2 (100%)	0 (0%)
East Asian-type		110 (98.2%)	45 (40.9%)	61 (55.5%)	4 (3.6%)
Pre-EPIYA					
18 bp deletion-type		94 (85.5%)	36 (38.3%)	54 (57.4%)	4 (4.3%)
39 bp deletion-type		13 (11.8%)	6 (46.2%)	7 (53.8%)	0 (0%)
No deletion-type		3 (2.7%)	1 (33.3%)	2 (66.7%)	0 (0%)
*vacA* genotype					
s region					
s1		111 (99.1%)	44 (39.6%)	63 (56.8%)	4 (3.6%)
s1s2		1 (0.9%)	1 (100%)	0 (0%)	0 (0%)
m region					
m1		77 (68.8%)	30 (39%)	45 (58.4%)	2 (2.6%)
m2		35 (31.2%)	15 (40.2%)	18 (51.4%)	2 (5.7%)

All *P* > 0.05

*n* number of cases

### Comparison of characteristics between the gastric cancer and non-cancer groups among infected patients

In our previous study, we recruited 270 participants from Cho Ray Hospital, Ho Chi Minh and 108 Military Hospital, Hanoi [28]. Among them, 103 cases were *H. pylori* culture-positive. Based on endoscopic observation, 26 PUD and 77 gastritis cases were diagnosed. GC and mucosa-associated lymphoid tissue (MALT) lymphoma were not detected in the study. We considered the data from the previous study to be a non-cancer group.

The average age of the GC group was significantly higher than that of the non-cancer group [28] (mean age, 61.7 vs 44.6, *P* < 0.0001). The difference in age between the two groups was also observed in both cities (*P* < 0.0001). The percentage of male patients within the GC group was significantly higher than within the non-cancer group (75.9% vs 45.6%, *P* < 0.0001). The same trend of male prevalence between cancer and non-cancer groups was observed in Ho Chi Minh, but not in Hanoi (*P* < 0.0001 and *P* = 0.064, respectively).

The prevalence of *H. pylori* infection in the GC group was significantly higher than in the non-cancer group (79.4% vs 66%, *P* = 0.0003). Univariate analysis showed that the *H. pylori* infected patients had a higher risk for non-cardia GC than did non-infected patients (OR, 2.02; 95% CI 1.4–3.0; *P* = 0.0003).

Similarly to our current data, our previous study also showed that most *H. pylori* isolates from non-cancer patients possessed the East Asian-type *cagA*. There were no differences in pre-EPIYA repeated region genotypes between the two groups. Likewise, most strains isolated from both cancer and non-cancer groups were the *vacA* s1 genotype (Table 3). Only the *vacA* m genotype showed a difference between isolates from cancer and non-cancer groups: a higher prevalence of *vacA* m1 was found in the cancer group than in the non-cancer group (68.8% vs 44.9%; *P* = 0.001). Univariate analysis showed that *vacA* m1 conferred an increased risk for non-cardia GC (OR, 2.7; 95% CI 1.5–4.7; *P* = 0.001). In Ho Chi Minh, the prevalence of *vacA* m1 within the cancer group was also significantly higher than that of the non-cancer group (67.6% vs 31.9%, *P* < 0.0001). In Hanoi, although *vacA* m1 was more prevalent in the cancer group than in the non-cancer group (70.7% vs 56.9%), the difference did not reach statistical significance (*P* = 0.17). Between the two cities, the prevalence of *vacA* m1 in Hanoi was higher than that in Ho Chi Minh; however the statistically significant difference between these two cities was only observed in the non-cancer group, but not in the cancer group (56.9% vs 31.9%, *P* = 0.023; 70.7% vs 67.6%, *P* = 0.83, respectively) (Table 3). Univariate analysis indicated that *vacA* m1 conferred an increased risk for non-cardia GC in Ho Chi Minh (OR, 4.5; 95% CI 2.0–9.8, *P* < 0.0001), but not in Hanoi (OR, 1.8; 95% CI 0.8–4.4, *P* = 0.17).

## Discussion

In Vietnam, GC remains the fourth most common type of cancer and is the third leading cause of cancer-related death in both genders (http://globocan.iarc.fr); therefore, GC is still an aggressive disease that continues to have a daunting impact on public health. This is the first study to describe the characteristics of the demographics and histopathology of non-cardia GC in the Vietnamese population. Consistent with many previous studies [30], our data in Vietnam showed that non-cardia GC has a late age of onset and a male preponderance. The incidence of non-cardia GC increases with age, with the peak mainly occurring after 50 years. These characteristics may reflect the underlying variation in *H. pylori* and lifestyle and environmental exposures.

Development of GC is a multistep and multifactorial process. The intestinal type is often related to *H. pylori* infection, diet, and lifestyle, while the diffuse type is more often associated with genetic abnormalities [31]. In this study, the overall proportion of diffuse carcinoma was found to be more predominant than the intestinal type, especially among younger patients and *H. pylori*-positive subjects. These results in Vietnam, which is a country with an intermediate risk of GC, are consistent with the global tendency, in which the intestinal type tends to predominate in geographic regions with a high incidence of GC and is less likely to be found in areas where the frequency is declining [32]. Moreover, all patients in this study were in the advanced stage of GC. This might be attributed to vague and non-specific symptoms in the early stages of GC and might cause the relatively poor prognosis of GC patients in Vietnam. In contrast, in Japan, due to the well-established strategy for GC prevention screening, most of the new cases are now diagnosed at an early stage. There, the patients prognosis is extremely good, with more than 90% surviving for 5 years or more [33]. With diffuse type preponderance in the study population, it reflects a younger trend in developing GC. Therefore, there is an urgent need to establish a screening guideline for GC, especially early GC in Vietnam.

The etiology of GC is multifactorial; however, *H. pylori* is attributable to about 70% in GC [34]. In this study, the serologic test showed the highest prevalence of *H. pylori* infection. Culture and histology were considered to be standard methods for detecting *H. pylori* because of its direct visibility. However, the two methods showed the lowest prevalence of infection. There are some possible explanations for this difference: first, *H. pylori* does not colonize areas affected by cancer, intestinal metaplasia, or atrophy, especially in advanced GC; secondly, there may be a bias of biopsy sampling areas. The advantage of serology is that both current and past infections can be detected. This means that even if *H. pylori* is lost from the stomach during the development of advanced GC, we can still obtain evidence of previous colonization of the organism for years. Although patients with history of *H. pylori* eradication were excluded, to avoid the bias of detection methodologies, we also analyzed current infection (at least two test positive) and past infection (only positive with serology test). However, there was no differences on age, sex, location, smoking, drinking, as well as Lauren classification types between these two groups. This is the reason why we did not apply the same criteria used in the previous study; *H. pylori*-positive status was determined by positive culture or, in the case of negative culture, by at least two positive results among remaining tests. Based on our current criteria, the prevalence of *H. pylori* infection was significantly higher in the cancer group than in the non-cancer group (79.4% vs 66%). In concordance with meta-analyses [35], our data showed that *H. pylori* infection was associated with approximately twofold increased risk of developing non-cardia GC. Preventative measures for GC have been developed with the focus on *H. pylori* therapy, and this has succeeded in decreasing the incidence as well as the mortality of GC [36–38]. The Asian Pacific gastric cancer consensus has recommended screening and eradication of *H. pylori* in communities with a high risk of GC (ASR > 20/100.000). The ASR of Vietnam is 16.3/100.000, which is near the standard threshold. With the burden of GC in Vietnam, an *H. pylori* eradication-based GC prevention strategy should be cost-effective and plausible.

*Helicobacter pylori* has emerged as the most important causal factor for GC; however, *H. pylori* infection alone is insufficient to cause GC. One hypothesis is that not all *H. pylori* strains are equal in virulence. Despite the similarity in ethnicity and dietary factors, the incidence of GC in Hanoi is 1.5 times higher than in Ho Chi Minh. In efforts to clarify this phenomenon, our previous study also partly proved the outstanding importance of virulence factors over the presence of bacteria in describing GC incidence in Vietnam [21]. Our results indicated that *vacA* m1, but not infection rate, correlated with an increased risk for PUD and contributed to the difference in the prevalence of GC between Hanoi and Ho Chi Minh.

Like several countries in East Asia where most strains harbored East Asian-type CagA regardless of the disease [39], the CagA presence failed to explain the differences in the spectrum of diseases of gastroenterology, including the CagA full-length sequences analysis (Additional file 1). In contrast, our previous study indicated that the occurrence of *vacA* m1 genotype of the non-cancer group in Hanoi was significantly higher than that in Ho Chi Minh (56.9% vs 31.9%) [21]. Nevertheless, the study only showed indirect evidence because of the lack of a comparison between GC and control cases. As a satisfactory answer to this concern, we first confirmed that there was a difference in the prevalence of *vacA* m sub-types between GC and non-cancer patients. Our current analysis showed that the occurrence of the *vacA* m1 genotype was significantly higher in the in the GC group than in the non-cancer group (68.8% vs 44.9%), and the significant tendency was still observed in Ho Chi Minh (67.6% vs 31.9%). It has been accepted that *vacA* m1 is more toxic than *vacA* m2, and individuals infected with *vacA* m1 strains have an increased risk of PUD and/or GC, compared to those infected with *vacA* m2 [19, 20]; therefore, our study strongly indicated that *vacA* m1 play an important role in GC in Vietnam, especially in Ho Chi Minh.

A limitation of this study is the difficulty of conducting a strong analytic, cross-sectional study. Because the number of cases of non-cardia GC is low, we conducted a long-term project for non-cardia GC subjects and compared those results with our previous study. Some information gathered in these two studies was not synchronous, which can cause some biases during interpretation. However, this study provides stronger evidence by using GC subjects.

In conclusion, we first described the characteristics of non-cardia GC in Vietnam late-age onset, predominance in males, and diffuse type. *H. pylori* infection was common and associated with the development of non-cardia GC. We also confirmed the association of the *vacA* m1 genotype with an increased risk of developing non-cardia GC, which might contribute to the difference in the incidence of non-cardia GC between Hanoi and Ho Chi Minh.

## Additional file

**Additional file 1.** Nucleotide sequence of full-length *cagA* and phylogenetic analysis.

## Abbreviations

GC: gastric cancer
*Helicobacter pylori*: *H. pylori*
PUD: peptic ulcer disease
ASR: age-standardized incidence rate
MALT: mucosa-associated lymphoid tissue

## References

[1] Ferro A, Peleteiro B, Malvezzi M, Bosetti C, Bertuccio P, Levi F, Negri E, La Vecchia C, Lunet N (2014). Worldwide trends in gastric cancer mortality (1980–2011), with predictions to 2015, and incidence by subtype. Eur J Cancer.

[2] Ang TL, Fock KM, Dhamodaran S, Teo EK, Tan J (2005). Racial differences in *Helicobacter pylori*, serum pepsinogen and gastric cancer incidence in an urban Asian population. J Gastroenterol Hepatol.

[3] Goh KL, Cheah PL, Md N, Quek KF, Parasakthi N (2007). Ethnicity and *H. pylori* as risk factors for gastric cancer in Malaysia: a prospective case control study. Am J Gastroenterol.

[4] Rahman R, Asombang AW, Ibdah JA (2014). Characteristics of gastric cancer in Asia. World J Gastroenterol.

[5] Piazuelo MB, Epplein M, Correa P (2010). Gastric cancer: an infectious disease. Infect Dis Clin North Am.

[6] Gonzalez CA, Agudo A (2012). Carcinogenesis, prevention and early detection of gastric cancer: where we are and where we should go. Int J Cancer.

[7] Peek RM, Blaser MJ (2002). *Helicobacter pylori* and gastrointestinal tract adenocarcinomas. Nat Rev Cancer.

[8] Suerbaum S, Michetti P (2002). *Helicobacter pylori* infection. N Engl J Med.

[9] Helicobacter, Cancer Collaborative G (2001). Gastric cancer and *Helicobacter pylori*: a combined analysis of 12 case control studies nested within prospective cohorts. Gut.

[10] Uemura N, Okamoto S, Yamamoto S, Matsumura N, Yamaguchi S, Yamakido M, Taniyama K, Sasaki N, Schlemper RJ (2001). *Helicobacter pylori* infection and the development of gastric cancer. N Engl J Med.

[11] Fox JG, Wang TC (2007). Inflammation, atrophy, and gastric cancer. J Clin Investig.

[12] Yamaoka Y (2010). Mechanisms of disease: *Helicobacter pylori* virulence factors. Nat Rev Gastroenterol Hepatol.

[13] Yamaoka Y, Kodama T, Kashima K, Graham DY, Sepulveda AR (1998). Variants of the 3′ region of the cagA gene in *Helicobacter pylori* isolates from patients with different H. pylori-associated diseases. J Clin Microbiol.

[14] Yamaoka Y, Orito E, Mizokami M, Gutierrez O, Saitou N, Kodama T, Osato MS, Kim JG, Ramirez FC, Mahachai V, Graham DY (2002). *Helicobacter pylori* in North and South America before Columbus. FEBS Lett.

[15] Jones KR, Joo YM, Jang S, Yoo YJ, Lee HS, Chung IS, Olsen CH, Whitmire JM, Merrell DS, Cha JH (2009). Polymorphism in the CagA EPIYA motif impacts development of gastric cancer. J Clin Microbiol.

[16] Vilaichone RK, Mahachai V, Tumwasorn S, Wu JY, Graham DY, Yamaoka Y (2004). Molecular epidemiology and outcome of *Helicobacter pylori* infection in Thailand: a cultural cross roads. Helicobacter.

[17] Azuma T, Yamakawa A, Yamazaki S, Ohtani M, Ito Y, Muramatsu A, Suto H, Yamazaki Y, Keida Y, Higashi H, Hatakeyama M (2004). Distinct diversity of the cag pathogenicity island among *Helicobacter pylori* strains in Japan. J Clin Microbiol.

[18] Leunk RD (1991). Production of a cytotoxin by *Helicobacter pylori*. Rev Infect Dis.

[19] Atherton JC, Cao P, Peek RM, Tummuru MKR, Blaser MJ, Cover TL (1995). Mosaicism in vacuolating cytotoxin alleles of *Helicobacter pylori* association of specific vacA types with cytotoxin production and peptic ulceration. J Biol Chem.

[20] Sugimoto M, Yamaoka Y (2009). The association of vacA genotype and *Helicobacter pylori*-related disease in Latin American and African populations. Clin Microbiol Infect.

[21] Nguyen TL, Uchida T, Tsukamoto Y, Trinh DT, Ta L, Mai BH, Le SH, Thai KD, Ho DD, Hoang HH, Matsuhisa T, Okimoto T, Kodama M, Murakami K, Fujioka T, Yamaoka Y, Moriyama M (2010). *Helicobacter pylori* infection and gastroduodenal diseases in Vietnam: a cross-sectional, hospital-based study. BMC Gastroenterol.

[22] Parkin DM, Whelan SL, Ferlay J, Teppo L, Thomas DB, editors. Cancer incidence in five continents, vol VIII. Lyon, France: IARC Scientific Publications; 2002. p. 1–781.

[23] Lauren P (1965). The two histological main types of gastric carcinoma: diffuse and so-called intestinal-type carcinoma. An attempt at a histo-clinical classification. Acta Pathol Microbiol Scand.

[24] Dixon MF, Genta RM, Yardley JH, Correa P (1996). Classification and grading of gastritis. The updated Sydney System. International Workshop on the Histopathology of Gastritis, Houston 1994. Am J Surg Pathol.

[25] Uchida T, Kanada R, Tsukamoto Y, Hijiya N, Matsuura K, Yano S, Yokoyama S, Kishida T, Kodama M, Murakami K, Fujioka T, Moriyama M (2007). Immunohistochemical diagnosis of the cagA-gene genotype of *Helicobacter pylori* with anti-East Asian CagA-specific antibody. Cancer Sci.

[26] Matsunari O, Shiota S, Suzuki R, Watada M, Kinjo N, Murakami K, Fujioka T, Kinjo F, Yamaoka Y (2012). Association between *Helicobacter pylori* virulence factors and gastroduodenal diseases in Okinawa, Japan. J Clin Microbiol.

[27] Yamaoka Y, El–Zimaity HMT, Gutierrez O, Figura N, Kim JK, Kodama T, Kashima K, Graham DY (1999). Relationship between the cagA 3′ repeat region of *Helicobacter pylori*, gastric histology, and susceptibility to low pH. Gastroenterology.

[28] Uchida T, Nguyen LT, Takayama A, Okimoto T, Kodama M, Murakami K, Matsuhisa T, Trinh TD, Ta L, Ho DQ, Hoang HH, Kishida T, Fujioka T, Moriyama M, Yamaoka Y (2009). Analysis of virulence factors of *Helicobacter pylori* isolated from a Vietnamese population. BMC Microbiol.

[29] Zhang XS, Tegtmeyer N, Traube L, Jindal S, Perez-Perez G, Sticht H, Backert S, Blaser MJ (2015). A specific A/T polymorphism in Western tyrosine phosphorylation B-motifs regulates *Helicobacter pylori* CagA epithelial cell interactions. PLoS Pathog.

[30] Karimi P, Islami F, Anandasabapathy S, Freedman ND, Kamangar F (2014). Gastric cancer: descriptive epidemiology, risk factors, screening, and prevention. Cancer Epidemiol Biomark Prev.

[31] Nagini S (2012). Carcinoma of the stomach: a review of epidemiology, pathogenesis, molecular genetics and chemoprevention. World J Gastrointest Oncol.

[32] Fuchs CS, Mayer RJ (1995). Gastric carcinoma. N Engl J Med.

[33] Miyahara R, Niwa Y, Matsuura T, Maeda O, Ando T, Ohmiya N, Itoh A, Hirooka Y, Goto H (2007). Prevalence and prognosis of gastric cancer detected by screening in a large Japanese population: data from a single institute over 30 years. J Gastroenterol Hepatol.

[34] de Martel C, Ferlay J, Franceschi S, Vignat J, Bray F, Forman D, Plummer M (2012). Global burden of cancers attributable to infections in 2008: a review and synthetic analysis. Lancet Oncol.

[35] Eslick GD (2006). *Helicobacter pylori* infection causes gastric cancer? A review of the epidemiological, meta-analytic, and experimental evidence. World J Gastroenterol.

[36] Ma JL, Zhang L, Brown LM, Li JY, Shen L, Pan KF, Liu WD, Hu Y, Han ZX, Crystal-Mansour S, Pee D, Blot WJ, Fraumeni JF, You WC, Gail MH (2012). Fifteen-year effects of *Helicobacter pylori*, garlic, and vitamin treatments on gastric cancer incidence and mortality. J Natl Cancer Inst.

[37] Ford AC, Forman D, Hunt RH, Yuan Y, Moayyedi P (2014). *Helicobacter pylori* eradication therapy to prevent gastric cancer in healthy asymptomatic infected individuals: systematic review and meta-analysis of randomised controlled trials. BMJ.

[38] Fukase K, Kato M, Kikuchi S, Inoue K, Uemura N, Okamoto S, Terao S, Amagai K, Hayashi S, Asaka M, Japan Gast Study G (2008). Effect of eradication of *Helicobacter pylori* on incidence of metachronous gastric carcinoma after endoscopic resection of early gastric cancer: an open-label, randomised controlled trial. Lancet.

[39] Yamaoka Y, Kodama T, Gutierrez O, Kim JG, Kashima K, Graham DY (1999). Relationship between *Helicobacter pylori* iceA, cagA, and vacA status and clinical outcome: studies in four different countries. J Clin Microbiol.

