# Supplementary material for: Advanced non-cardia gastric cancer and Helicobacter pylori infection in Vietnam
Source: Gut Pathog. 2017 Aug 17;9:46. doi: 10.1186/s13099-017-0195-8 (PMC5561603; doi:10.1186/s13099-017-0195-8)
Supplement: Supplementary file 1 — Additional file 1. Nucleotide sequence of full-length cagA and phylogenetic analysis. [file 13099_2017_195_MOESM1_ESM.pdf]

Nucleotide sequence of full-length *cagA* and phylogenetic analysis

The full-length *cagA* sequences were determined as described previously [1]. For phylogenetic analysis of *cagA* sequences, 14 East-Asian-type *cagA* strains from gastric cancer (GC) patients and 14 East-Asian-type *cagA* strains from gastritis patients were randomly selected **(Table S1**) with Genbank accession numbers from LC314739 to LC314766; the other strains from surrounding countries such as Thailand, Cambodia, Philippines, China, Japan (mainland and Okinawa prefecture), and several Western countries were also obtained from GenBank as reference sequences. The GenBank accession numbers are as follows: Thailand, GU173873–GU173875, GU173877–GU173879; Cambodia, JF798688–JF798692, JF798698–JF798706; the Philippines, GU173858–GU173861, GU173864–GU173867, GU173869–GU173872; China, AF249275, AF367250, EU681369; Japan (mainland), AB090075, AB190964, AB190935, AB017921–AB017923, AB015411, AB015412; Japan (Okinawa), AB190937, AB190940–AB190943, AB190946–AB190948, AB190950, AB190951, AB190954, AB190956, AB190968, AB246735, AB246737; and Western countries, 26695, ATCC49503-W, ATCC43579-W, ATCC43526-W, NCTC11637-Autralia. The full-length *cagA* sequences of strains from GC patients and reference sequences were aligned using CLUSTALW program in MEGA 6.0 software (Molecular Evolutionary Genetics Analysis, Tempe, AZ, USA). Phylogenetic tree was constructed using the neighbor-joining method, and bootstrap test (1,000) for phylogeny was performed using MEGA 6.0.

Figure legend for Figure S1

The phylogenetic tree of *cagA* full-length sequences showed that the strains isolated from GC and chronic gastritis in Vietnam were scattered into the same branch like the other East-Asian- *cag*A strains.

Table S1 Strains isolated from 14 gastric cancer vs 14 gastritis

Strains (n=28)	Diseases	EPIYA Segment type	full-length *cagA* (bp)	Protein (aa)	Homology with East-Asian type strain F32 (%)	Homology with Western type strain 26695 (%)
CHC06	Gastric cancer	ABD	3546	1182	94.3	86.6
CHC38		ABD	3546	1182	94.4	86.3
CHC40		ABD	3546	1182	94.7	86.1
CHC62		ABD	3531	1177	95.9	86.5
CHC65		ABD	3546	1182	94.7	86.5
CHC66		ABD	3549	1183	94.6	86.7
CHC68		ABD	3531	1177	95.0	86.5
CHC74		ABD	3555	1185	95.2	86.9
CHC77		ABD	3546	1182	94.7	86.6
CHN08		ABD	3549	1183	95.3	86.0
CHN12		ABD	3543	1181	94.4	86.3
CHN20		ABD	3543	1181	94.3	86.1
CHN22		ABD	3585	1195	95.0	86.6
CHN36		ABD	3528	1176	96.4	86.3
HC029	Gastritis	ABD	3543	1181	94.6	86.0
HC034		ABD	3537	1179	95.9	86.0
HC038		ABD	3540	1180	95.3	86.7
HC061		ABD	3528	1176	94.2	86.8
HC093		ABD	3546	1182	94.9	86.4
HC124		ABD	3603	1201	92.5	85.4
HC132		ABD	3528	1176	95.2	85.9
HC150		ABD	3543	1181	94.9	86.4
HN021		ABD	3546	1182	94.7	86.1
HN031		ABD	3552	1184	94.5	86.5
HN073		ABD	3552	1184	95.0	86.6
HN111		ABD	3546	1182	94.9	86.3
VH010		ABD	3543	1181	94.7	86.2
VH133		ABD	3546	1182	94.6	86.3

Figure S1 Phylogenetic tree based on full-length *cagA* sequences of strains isolated from 14 gastric cancer vs 14 gastritis

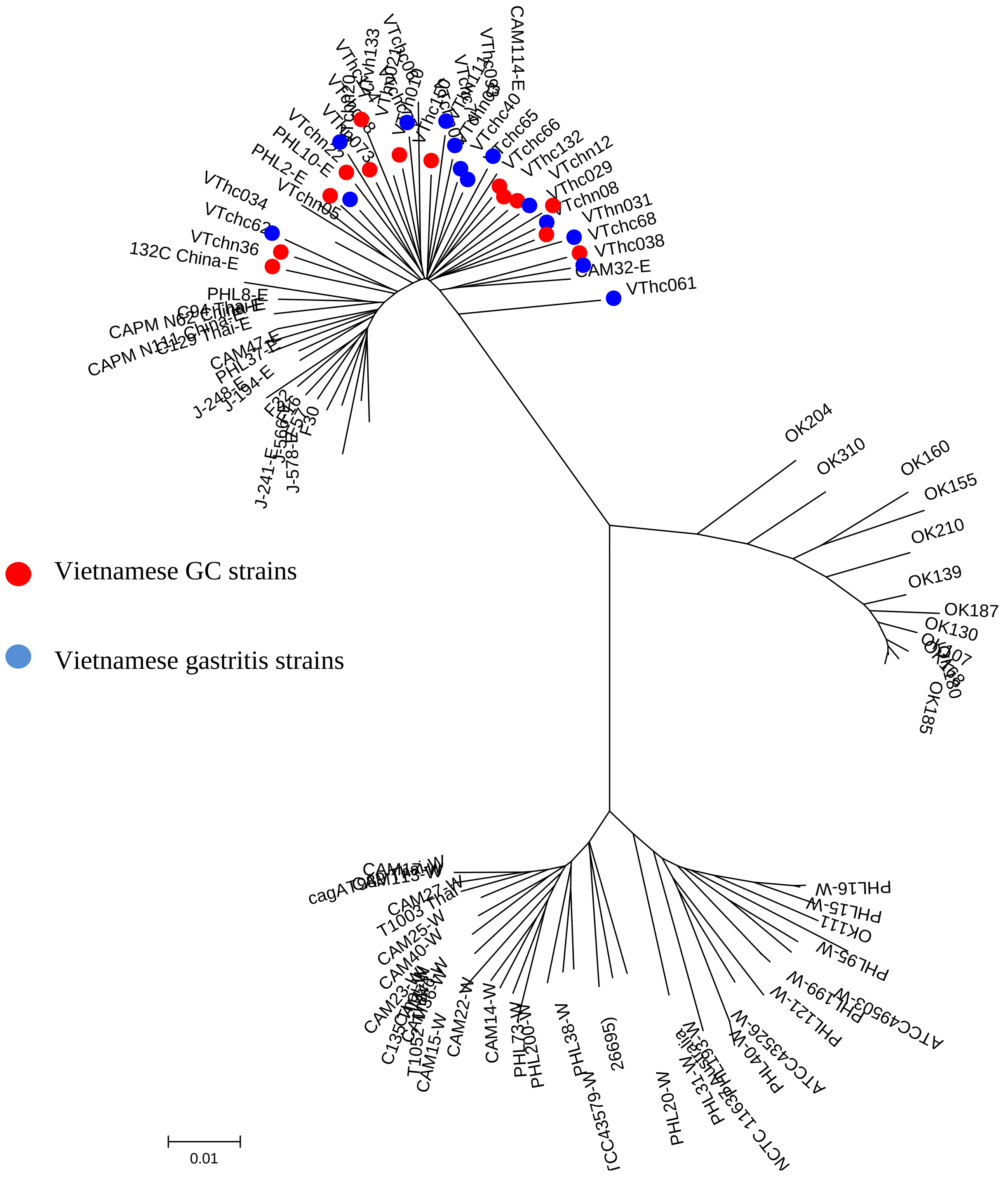

Reference

1. Cortes MC, Yamakawa A, Casingal CR, Fajardo LS, Juan ML, De Guzman BB, Bondoc EM, St. Luke's Helicobacter pylori Study G, Mahachai V, Yamazaki Y, Yoshida M, Kutsumi H, Natividad FF, Azuma T. Diversity of the cagA gene of *Helicobacter pylori* strains from patients with gastroduodenal diseases in the Philippines. *FEMS Immunol Med Microbiol.* 2010; 60:90-97. doi: 10.1111/j.1574-695X.2010.00722.x
